# AMPK is not required for the effect of metformin on the inhibition of BMP6-induced hepcidin gene expression in hepatocytes

**DOI:** 10.1038/s41598-017-12976-2

**Published:** 2017-10-04

**Authors:** Jean-Christophe Deschemin, Marc Foretz, Benoit Viollet, Sophie Vaulont

**Affiliations:** 1 INSERM, U1016, Institut Cochin, Paris, France; 2 CNRS, UMR8104 Paris, France; 3 Université Paris Descartes, Sorbonne Paris Cité, Paris, France; 4 Laboratory of Excellence GR-Ex, Paris, France

## Abstract

The biguanide metformin is used for its antidiabetic effect for many years but how metformin acts remains poorly understood and controversial. AMP-activated protein kinase (AMPK), a protein kinase that plays a key role in maintaining energy homeostasis, is assumed to be one of the prime targets of metformin. However, since our demonstration that AMPK is not required for the beneficial effects of metformin on the control of glycemia, the list of AMPK-independent actions of metformin is rapidly increasing. Given the conflicting results on the effects of metformin we sought, using our genetic mouse models deficient in the catalytic subunits of AMPK, to determine whether this kinase is involved in the effects of metformin on the expression of the iron-regulatory hormone hepcidin, as recently proposed. Here we demonstrate, using different approaches, either isolated hepatocytes that lack AMPK, or direct AMPK activators, that, AMPK activation is not necessary for metformin to inhibit BMP6-induced hepcidin gene expression. These results may shed new lights on the increasingly recognized AMPK-independent metformin’s molecular action, an important area of current research.

## Introduction

In a recent study, Kim *et al*. reported an interesting finding of transcriptional regulation of the iron-regulatory hormone hepcidin expression by the antidiabetic biguanide drug metformin^1^. Hepcidin plays a critical role in the control of systemic iron balance by coordinating the major fluxes of iron into blood plasma through the regulation of intestinal iron absorption, delivery of recycled iron from macrophages, and release of stored iron from hepatocytes^2^. It is known that dietary iron regulates hepcidin expression through BMP6-mediated activation of SMAD1/5/8 signaling^3^. Kim *et al*. showed that metformin treatment blunted the BMP6-controlled hepcidin response and attenuated the BMP6-related changes of iron metabolism in mice. While the detailed mechanisms underlying the effects of metformin have not been completely elucidated, there is prior evidence that its primary effect is in mitochondria, where the drug interferes with respiratory complex I and reduces ATP production^4,5^, leading to the activation of AMP-activated protein kinase (AMPK)^6^, a protein kinase that has a key role in maintaining energy homeostasis^7^.

In view of their previous observation that expression of the small heterodimer partner (SHP; NR0B2), an atypical orphan member of the nuclear receptor superfamily^8^, was strongly induced by metformin through AMPK activation^9^, the authors further tested the hypothesis that AMPK activation and subsequent SHP expression could affect BMP6 signaling-induced hepcidin expression. In line with this hypothesis, the authors indeed showed that metformin treatment suppresses BMP6-mediated hepcidin response both in hepatoma cells and primary hepatocytes, a suppressive effect that was attenuated by either SHP knockdown, treatment with a non specific inhibitor of AMPK (Compound C, also known as dorsomorphin) or a dominant negative form of AMPK. They therefore concluded that AMPK signaling is connected to the regulation of iron metabolism through induction of SHP and control of hepcidin gene expression.

Since the precise mechanism of action of metformin remains controversial, with activation of both AMPK-dependent and AMPK-independent pathways^10^, we were interested to further investigate the putative involvement of AMPK in suppression of BMP6-mediated hepcidin up-regulation by metformin.

## Methods

All methods were carried out in accordance with relevant guidelines and regulations.

### Animals

All procedures were performed in accordance with the principles and guidelines established in the European Convention for the Protection of Vertebrate Animals Used for Experimental and Other Scientific Purposes (Council of Europe, ETS no. 123, 1991). Animal studies described herein were reviewed and approved (agreement no. 75–886) by the Directeur Départemental des Services Vétérinaires of the Préfecture de Police de Paris. Mice lacking both AMPKα1 and AMPKα2 catalytic subunits in the liver were previously described^11–13^. Unless challenged in conditions inducing changes in the ATP/AMP ratio (such as pharmacological-induced energy stress^11,12^ or nutrient deprivation^13^), these mice presented no metabolic defect in basal conditions.

Littermates from the same breeding pair were used in these experiments. Hepatocytes were isolated and cultured as described^11^. Briefly, hepatocytes were isolated from fed adult male mice of 8–10 week-old by the collagenase method. The cells were plated in M199 medium supplemented with 10% FCS. After attachment (3–4 hours), hepatocytes were maintained in M199 medium for additional 24 h in presence of 0.1% FCS. Medium was changed and AMPK activators (0.25 to 1 mM metformin, 3 μM 991 or 3 μM C13) were added for 1 h prior supplemental 24 h of culture in presence of 10 nM BMP6.

### Reverse transcription and real-time quantitative PCR

RNA extraction, reverse transcription and quantitative PCR were performed as previously described^14^. All samples were normalized to the threshold cycle value for cyclophilinA.

### Western blot

Total lysate extracts from hepatocytes were prepared as described^11^. Briefly, hepatocytes were lysed in ice-cold lysis buffer and sonicated on ice. The homogenate was centrifuged for 10 minutes at 10000 *g* at 4 °C and the supernatants were removed for determination of total protein content. Twenty micrograms of proteins from the supernatant was separated on 10% SDS-PAGE gels and transferred onto nitrocellulose membranes. Immunoblotting was performed following standard procedures, and the signals were detected by chemiluminescence reagents. Primary antibodies were directed against total AMPKα, AMPKα phosphorylated at Thr172, total ACC and ACC phosphorylated at Ser79 (from Cell Signaling Technology).

### Statistical analysis

Statistical analysis was performed using the Newmans-Keuls test. *P* values less than .05 were considered statistically significant.

## Results and Discussion

**Figure 1:**
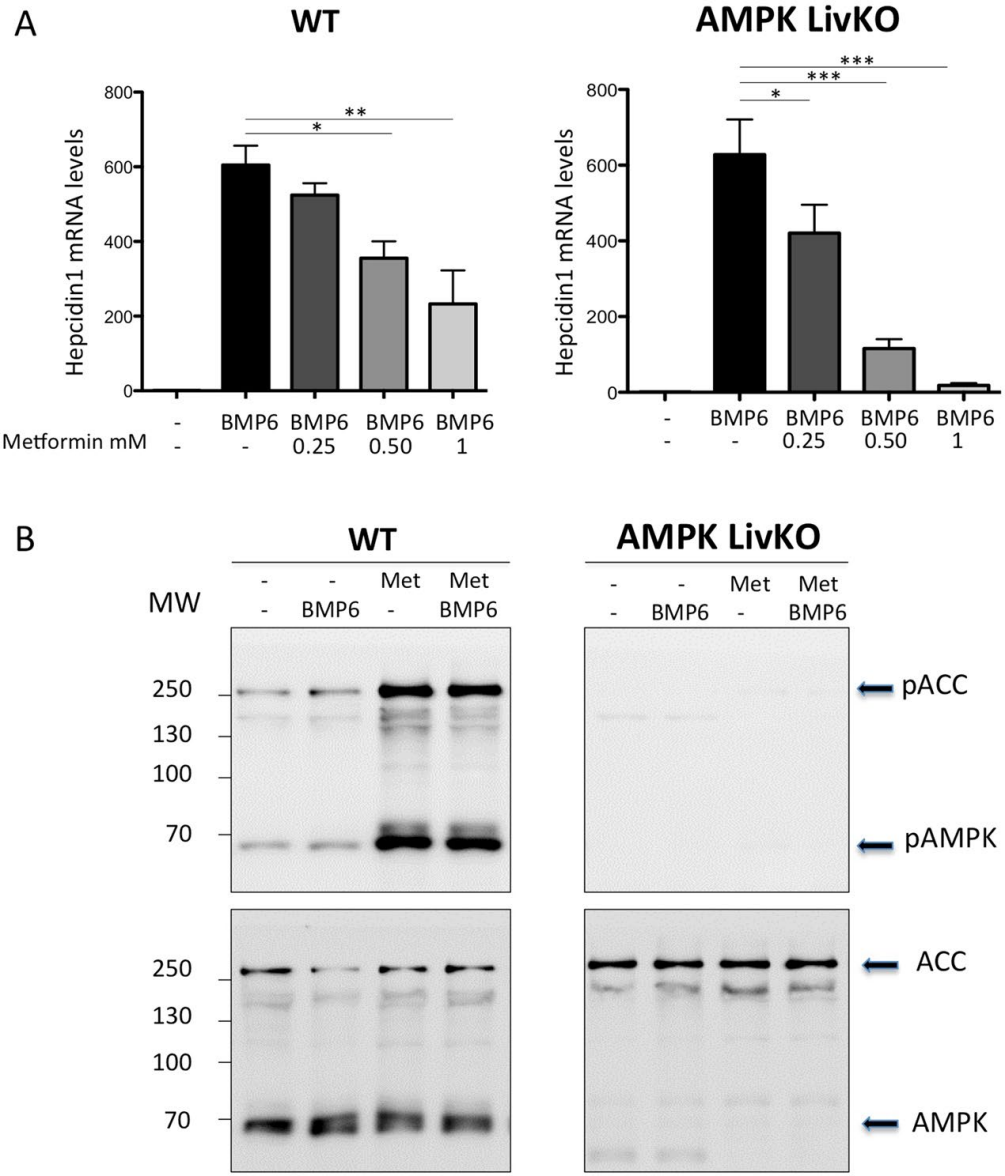
Effect of metformin on BMP6-induced hepcidin gene expression in WT and AMPKLiv KO mice.(**A**) Q-PCR analysis showing hepcidin mRNA levels in mouse primary hepatocytes treated with BMP6 (10 nM) or/and metformin (from 0.25 to 1 mM) for 24 h. RNA extraction and real-time quantification of transcripts were performed as described^14^. mRNA expression was calculated using the ΔΔCt method and normalized to the expression of cyclophilin A. Hepcidin gene expression in BMP6 treated hepatocytes, with or without metformin, is expressed as-fold hepcidin expression of that in non-treated hepatocytes. Error bars represent SD for n = 3 samples in each group. Statistical significance is indicated by * (*p < 0.05, **p < 0.005, ***p < 0.0005 as compared to the BMP6 condition). A typical representative experiment is shown. Similar results were confirmed in three separate experiments. (**B**) Immunoblots showing pAMPK/AMPK and pACC/ACC in hepatocyte extracts prepared as previously described, treated either with 10 nM BMP6, 1 mM metformin or both.

To determine the role of AMPK on the effect of metformin on hepcidin activation by BMP6, we isolated and treated hepatocytes from WT and AMPK livKO. Importantly, at 2 mM metformin, we noticed a strong toxic effect of the drug leading to a high mortality rate of the hepatocytes after 24 h of culture. We thus decided to work with metformin concentrations ranging from 0.25 to 1 mM. Here, we confirmed in the WT hepatocytes that metformin acts in a dose- dependent manner to inhibit BMP6-mediated hepcidin gene expression. However, strikingly, an even stronger repression was observed in AMPK- deficient hepatocytes (Fig. 1A), clearly demonstrating that metformin-induced repression of hepcidin gene expression is not mediated through AMPK activation. As expected, metformin efficiently stimulated AMPK activation as illustrated by increased phosphorylation of AMPK at Thr172 and of its well-established substrate, acetyl-CoA carboxylase (ACC) in WT hepatocytes only (Fig. 1B).

**Figure 2:**
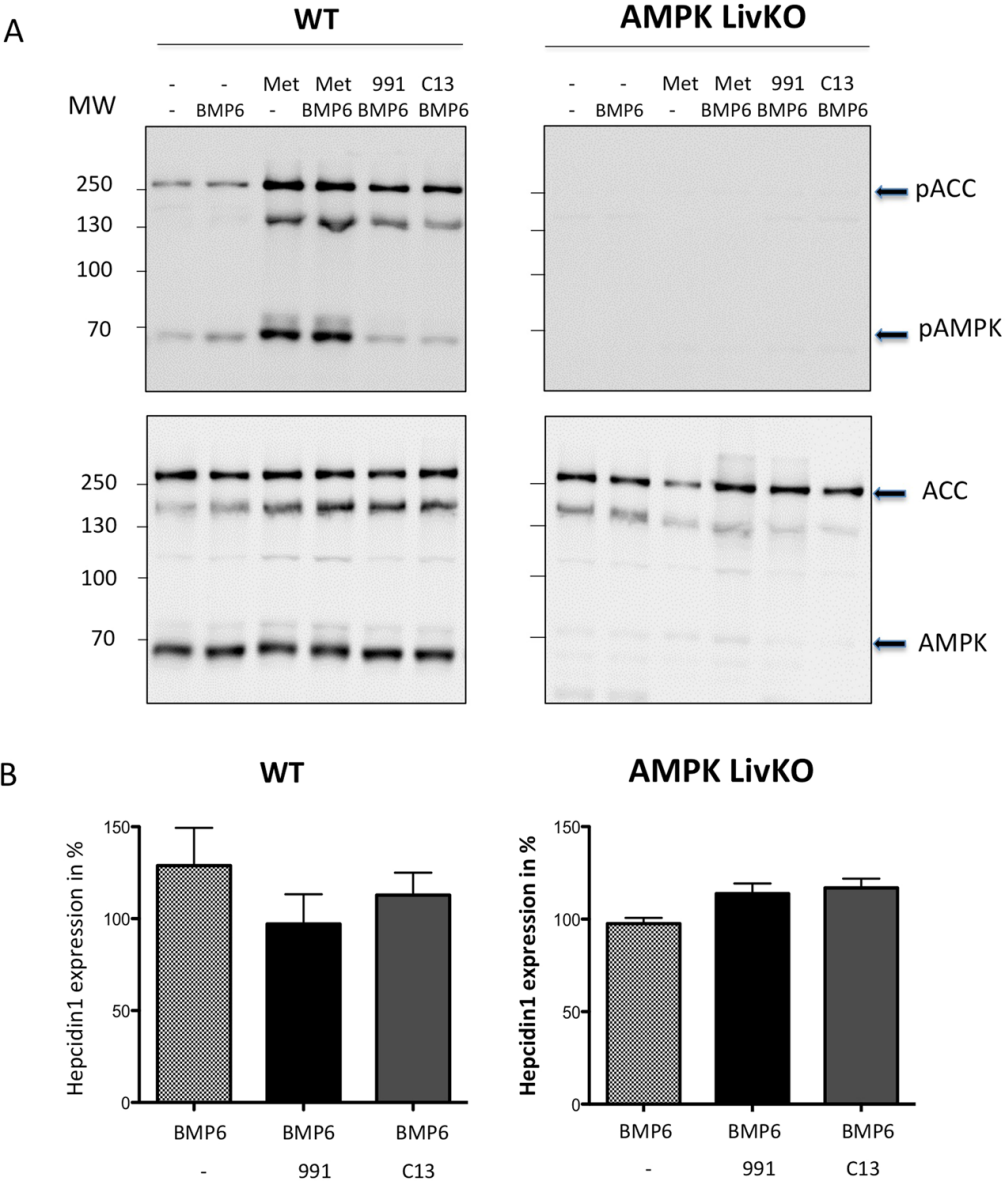
Effect of 991 and C13 on BMP6-induced hepcidin gene expression in WT and AMPKLiv KO mice. (**A**) Immunoblots showing pAMPK/AMPK and pACC/ACC in hepatocyte extracts prepared as previously described, treated with 10 nM BMP6, 3 μM 991 or 3 μM C13. (**B**) Q-PCR analysis showing hepcidin mRNA levels in mouse primary hepatocytes treated with BMP6 (10 nM), 991 (3μM) or C13 (3μM) for 24 h. RNA extraction and real-time quantification of transcripts were performed as described. mRNA expression was calculated using the ΔΔCt method and normalized to the expression of cyclophilin A. Hepcidin gene expression is expressed in % of BMP6 treated hepatocytes. ﻿Error bars represent SD for n = 3 samples in each group.

To further demonstrate the lack of AMPK-mediated regulation of hepcidin gene expression, we tested the effect of two direct allosteric AMPK activators, compound 991, a small-molecule benzimidazole derivative^15^ and compound C13^16^, whose activities were shown to be largely ineffective in AMPKLiv KO hepatocytes^16,17^. As previously reported, at 3 µM, 991 and C13 were ineffective in increasing AMPK phosphorylation in WT primary hepatocytes^16,18^, but a robust increase in ACC phosphorylation was readily observed, indicating AMPK activation (Fig. 2A). However, while AMPK is well activated by these small molecule activators, they were inefficient in inhibiting hepcidin gene expression in response to BMP6 treatment, whatever the genotype of the mice (Fig. 2B). Noteworthy, none of the AMPK activators tested (metformin, 991 and C13) were found to alter hepcidin gene expression in absence of BMP6 (Supp. Fig. 1).

**Figure 3:**
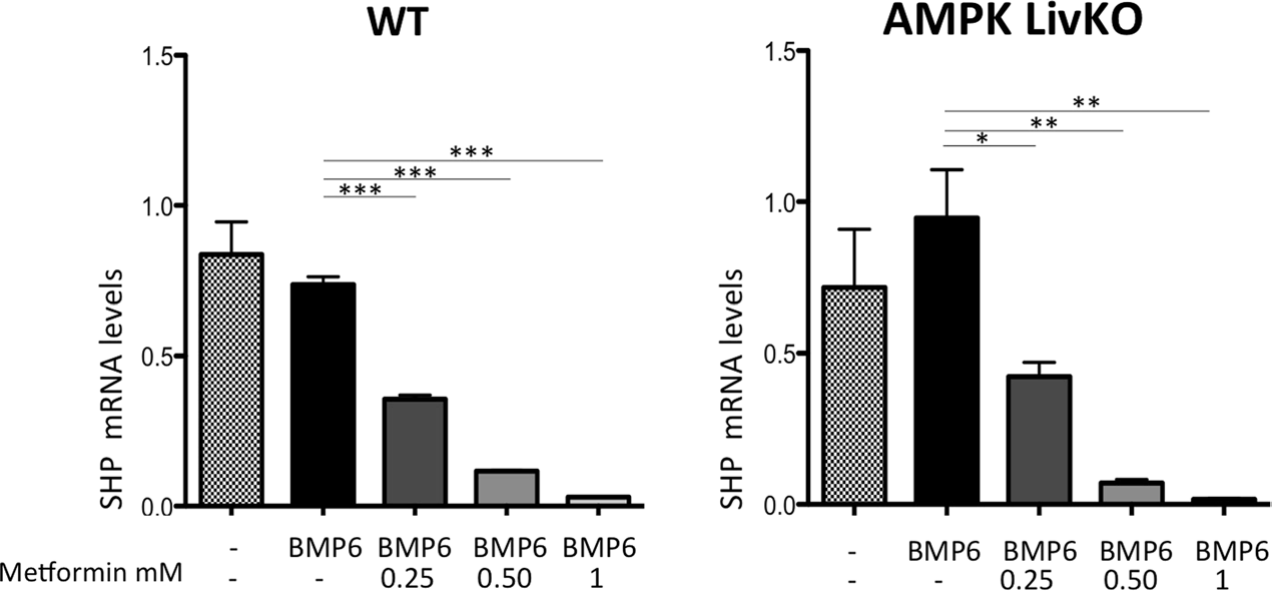
Effect of metformin on BMP6-mediated SHP gene expression in WT and AMPKLiv KO mice. (**A**) Q-PCR analysis showing SHP mRNA levels in mouse primary hepatocytes treated with BMP6 (10 nM) or/and metformin (from 0.25 to 1 mM) for 24 h. RNA extraction and real-time quantification of transcripts were performed as described. mRNA expression was calculated using the ΔΔCt method and normalized to the expression of cyclophilin A. SHP gene expression in BMP6 treated hepatocytes, with or without metformin, is expressed as-fold SHP expression of that in non-treated hepatocytes. Error bars represent SD for n = 3 samples in each group. Statistical significance is indicated by * (*p < 0.05, **p < 0.005, ***p < 0.0005 as compared to the BMP6 condition).

In view of this AMPK- independent effect of metformin, we questioned the role of SHP in the repression of hepcidin, SHP being supposedly induced by AMPK activation. Surprisingly and hardly reconcilable with such a role for SHP, we found a drastic dose- dependent reduction of SHP mRNA levels after treatment with metformin, regardless of the presence of BMP6 (Fig. 3 and Supp. Fig. 2), in contrast to the data reported by Kim *et al*.^1^. Noteworthy, our results are consistent with previous observations showing a repression of SHP mRNA levels by metformin in mouse hepatocytes^19^. In addition, Krausova *et al*. did not detect any increase in SHP mRNA levels in response to metformin in human primary hepatocytes^20^ and observed a significant down regulation in the MZ-Hep1 hepatoma cell line incubated for 4 h with 2 mM metformin.

There is today an abundant literature pointing out AMPK- independent effects of metformin. Metformin is currently the drug of first choice for the treatment of type 2 diabetes for its glucose-lowering effect^10^. By using AMPKLiv KO mice and hepatocytes, we established for the first time that AMPK is not required for metformin-mediated inhibition of hepatic glucose production and gluconeogenic gene expression^11^. We proposed that a decreased of cellular energy charge (concomitant decrease in ATP and increase in AMP intracellular levels) resulting from metformin’s inhibition of mitochondrial respiratory-chain complex I^4,5^ was a plausible explanation of its AMPK-independent action^11,21^. Hence, metformin-dependent deficit in ATP levels reduces the energy-demanding process of gluconeogenesis. In addition, the concomitant increase in AMP levels functions as a key signalling mediator to allosterically inhibit the gluconeogenic enzyme fructose-1,6-bisphosphatase and also cAMP–PKA signalling through suppression of adenylate cyclase^10^. More recently, Madiraju *et al*. proposed that the reduction of gluconeogenesis evoked by metformin may be a result of increased cytosolic redox state and decreased mitochondrial redox state elicited by direct inhibition of mitochondrial glycerophosphate dehydrogenase^22^.

Apart from these metabolic effects of metformin, it is now well recognized that AMPK is also dispensable for some of the other pleiotropic effects of the drug. To name a few, we can mention the antineoplastic potential of metformin shown to be mediated through REDD1, a negative regulator of mTOR^23^. Along with these studies, metformin was also reported to down-regulate mTORC1 through the RAG family of GTPases^24^. Recently, Cameron *et al*. demonstrated that metformin treatment suppressed TNFα-induced degradation of the NF-κB negative regulator IκB and that AMPK was not required for these effects in the liver^25^.

In conclusion, our data on the effect of metformin on hepcidin cannot reliably support the conclusion that metformin-mediated repression of BMP6-induced hepcidin gene expression is mediated through an AMPK/SHP axis. Further analyses are now necessary to determine the precise role of SHP and whether identified alternative pathways, such as the ones discussed here, or novel ones, are required for the effect of metformin.

## Electronic supplementary material

Supplementary Information
