# Supplementary material for: AMPK is not required for the effect of metformin on the inhibition of BMP6-induced hepcidin gene expression in hepatocytes
Source: Sci Rep. 2017 Oct 4;7:12679. doi: 10.1038/s41598-017-12976-2 (PMC5627262; doi:10.1038/s41598-017-12976-2)
Supplement: Supplementary file 1 — Supplementary Information [file 41598_2017_12976_MOESM1_ESM.pdf]

AMPK is not required for the effect of metformin on the inhibition of BMP6-induced hepcidin gene expression in hepatocytes.

Jean-Christophe Deschemin [1,2,3,4,*], Marc Foretz [1,2,3,4,*], Benoit Viollet [1,2,3,4] and Sophie Vaulont [1,2,3,4,$]

[1] INSERM, U1016, Institut Cochin, Paris, France
[2] CNRS, UMR8104, Paris, France
[3] Université Paris Descartes, Sorbonne Paris Cité, Paris, France
[4] Laboratory of Excellence GR-Ex
[*] Equal contribution

[$] To whom correspondence should be addressed:
Dr Sophie Vaulont, Institut Cochin, U1016,
24 Rue du Fg St Jacques, 75014, Paris, France
Telephone: 001 403 210 3938; Fax: 001 403 270 8928, e-mail : sophie.vaulont@inserm.fr

Legend of Figures

Supplemental Figure 1: Effect of metformin, 991 and C13 on basal hepcidin gene expression in WT and AMKPLiv KO mice.

(A) Q-PCR analysis showing hepcidin mRNA levels in mouse primary hepatocytes treated with metformin (from 0.25 to 1mM) for 24 h), 991 (3μM) or C13 (1μM) for 24 h. RNA extraction and real-time quantification of transcripts were performed as described. mRNA expression was calculated using the ΔΔCt method and normalized to the expression of cyclophilin. Hepcidin gene expression is expressed as-fold hepcidin expression of that in non-treated hepatocytes. Error bars represent SD for n=3 samples in each group.

Supplemental Figure 2: Effect of metformin on basal SHP gene expression in WT and AMPKLiv KO mice.

(A) Q-PCR analysis showing SHP mRNA levels in mouse primary hepatocytes treated with metformin (from 0.25 to 1mM), 991 (3μM) or C13 (3μM) for 24 h. RNA extraction and real-time quantification of transcripts were performed as described. mRNA expression was calculated using the ΔΔCt method and normalized to the expression of cyclophilin. SHP gene expression is expressed as-fold SHP expression of that in non-treated hepatocytes.

Error bars represent SD for n=3 samples in each group. Statistical significance is indicated by * symbols (*p< 0.05, **p< 0.005, ***p< 0.0005 as compared to that of non-treated hepatocytes).

SFig 1

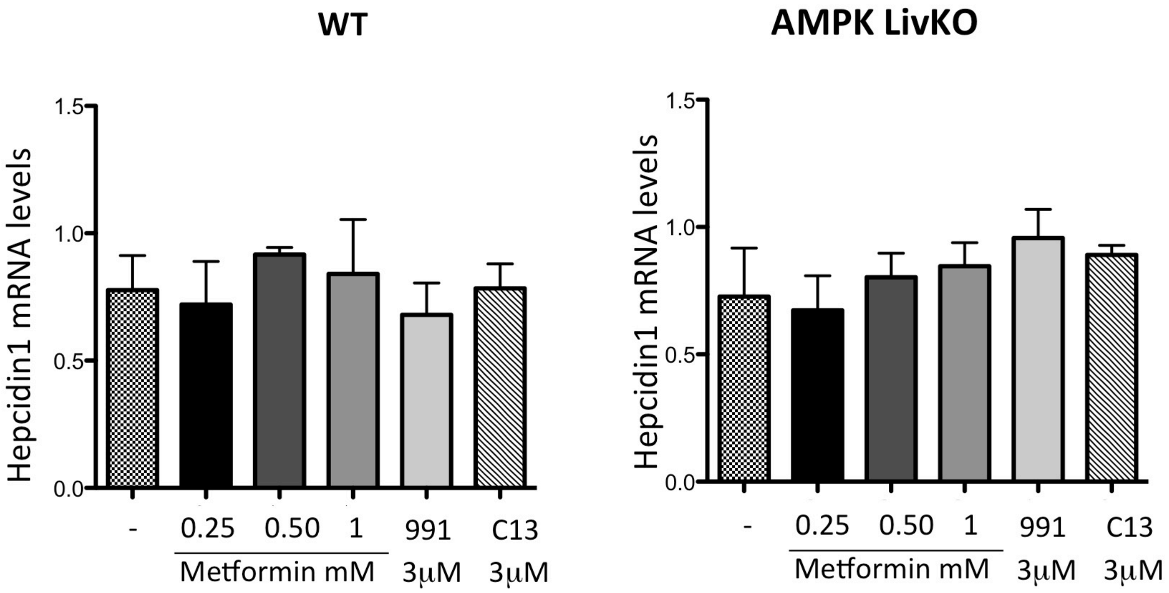
WT
AMPK LivKO
Hepcidin1 mRNA levels
1.5
1.0
0.5
0.0
-
0.25
0.50
1
991
C13
Metformin mM
3μM
3μM

SFig 2

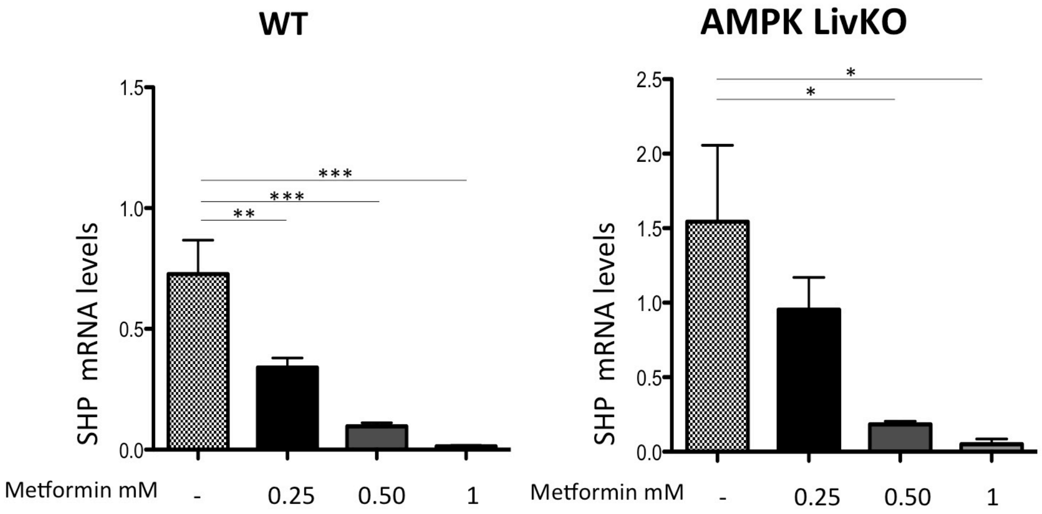
WT
AMPK LivKO
SHP mRNA levels
1.5
1.0
0.5
0.0
2.5
2.0
***
**
*
Metformin mM
-
0.25
0.50
1
